# Cleaning products and classes associated with poor respiratory health

**DOI:** 10.1007/s11356-026-37616-z

**Published:** 2026-03-23

**Authors:** Xin Dai, Michael J. Abramson, Garun S. Hamilton, Bruce R. Thompson, Cecilie Svanes, Geza Benke, Sonia Kaushik, Shyamali C. Dharmage, Caroline J. Lodge

**Affiliations:** 1 Allergy and Lung Health Unit, Centre for Epidemiology and Biostatistics, Melbourne School of Population and Global Health, The University of Melbourne, 207 Bouverie Street, Carlton, VIC 3010 Australia; 2 School of Public Health and Preventive Medicine, Monash University, Melbourne, VIC Australia; 3 Department of Lung, Sleep, Allergy and Immunology, Monash Health, Clayton, VIC Australia; 4 School of Clinical Sciences, Monash University, Clayton, VIC Australia; 5 Faculty of Medicine, Dentistry and Health Sciences, Melbourne School of Health Sciences, The University of Melbourne, Carlton, VIC Australia; 6 Centre for International Health, Department of Global Public Health and Primary Care, University of Bergen, Bergen, Norway; 7 Department of Occupational Medicine, Haukeland University Hospital, Bergen, Norway

**Keywords:** Questionnaires, Cleaning products, Poor respiratory health, Asthma, Lung function, COPD, Cross-sectional study

## Abstract

Exposure to cleaning products may harm the lungs, mainly through inhalation of irritants and sensitising chemicals, which can induce airway inflammation and bronchial hyperresponsiveness. Given increased use of multiple cleaning products at work and home, understanding the impacts of their interplay, rather than individual exposures, is critical but has not been investigated to date. We aim to investigate the cross-sectional association between exposure to cleaning products at home and/or in the workplace and respiratory health. We conducted a cross-sectional analysis of 318 adults from the Melbourne arm of the European Community Respiratory Health Survey (ECRHS) III. Cleaning product exposure was assessed through questionnaires, categorising participant exposure into seven product groups. Latent class analysis was used to identify exposure classes. Adjusted multivariable regression modelled associations between cleaning product classes and respiratory outcomes. We identified four classes of exposure to cleaning products: “minimal users”, “light users”, “moderate users”, “heavy users”. The most exposed “heavy user group” characterised people using many different cleaning products on a weekly basis (especially bleach, sprays, polish, solvents, acids). This class was associated with increased risks of current asthma (OR, 3.24; 95% CI 1.19–8.77), and lower post-bronchodilator FEV_1_ (z-score, −0.47) and FVC (−0.46) compared with “minimal users”. This work used a data-driven latent class approach to capture real-world cleaning product use patterns and relate them to respiratory health. We found that frequent use of multiple cleaning products was linked to more asthma and lower lung function, suggesting potential combined effects. These findings highlight the need for cleaning product standards and asthma care guidelines to mitigate risks associated with cleaning products.

**Supplementary information:**

The online version contains supplementary material available at 10.1007/s11356-026-37616-z.

## Introduction

The COVID-19 pandemic has heightened global awareness of hygiene practices, leading to increased use of cleaning products in homes and public spaces (Vayisoglu and Oncu 2021). These products include general sprays, acids, bleach, ammonia, degreasers, and other chemicals. Evidence on the safety of cleaning products for respiratory health is largely based on occupational exposures (Vizcaya et al. 2015; Mwanga et al. 2023; Lee et al. 2024; Annett et al. 2025). Exposure to chemical agents may irritate the lungs and cause inflammation, leading to poor respiratory health among occupationally exposed workers (Vizcaya et al. 2015; Dumas et al. 2019; Dang et al. 2022; Patel et al. 2024). Current asthma and chronic obstructive pulmonary disease (COPD) management guidelines rarely mention the safety of cleaning products used in the home (Global Initiative for Asthma 2024; National Asthma Council 2025), as there is currently limited evidence on commonly used cleaning products in daily life.

Cleaning products contain complex ingredients, varying according to their type (sprays, liquids, foams) and use (multipurpose, kitchen surfaces, bathrooms, glass, polishing, etc.) (Lemire et al. 2022). Commonly, multiple cleaning products are used concurrently at home and work, potentially leading to combined or amplified respiratory effects. Previous studies have primarily focused on individual ingredients or specific products (Zock et al. 2007; Casas et al. 2013; Le Moual et al. 2014; Ersanli and Berktas 2025), which may not fully capture the risks posed from the use of mixtures of products for everyday cleaning practices. Latent class analysis (LCA) offers a data-driven approach to characterise patterns of multiple cleaning product use by identifying groups of individuals with similar exposure profiles (Da Silva et al. 2025). Our approach may better reflect real-world exposure scenarios compared with traditional single-exposure analyses.

We collected data from participants in the Melbourne arm of the third wave of the European Community Respiratory Health Survey (ECRHS III), aiming to identify patterns of cleaning product used at home and work using LCA, and to examine cross-sectional associations with asthma, COPD, and lung function among adults.

## Methods

### Setting and participants

The European Community Respiratory Health Survey (ECRHS) is a large, multicentre, international research project initiated in the early 1990 s to investigate the prevalence, risk factors, and long-term health outcomes of asthma (Zock et al. 2007; Svanes et al. 2018). The Australian centre collected data from 754 participants residing in the southeastern Melbourne region, Victoria, Australia, including a random general adult population (*N* = 552) and a symptomatic sample (*N* = 202), with multiple follow-ups to track changes in respiratory health over time. We used cross-sectional data from the third Melbourne follow-up (2010–2012), that recruited 550 participants in total. Of them, the analytic sample comprised 318 completing the main questionnaire for asthma. Analyses of lung function outcomes were restricted to 277 participants undergoing clinical tests for lung function. Ethic approval for this study was obtained from Monash University Human Research Ethics Committee (Project number: CF11/1818–2011001012) on 27th September 2011. Participants gave written consent to participate in the study before taking part. Clinical trial number is not applicable for this study. Further details of the ECRHS follow-up procedure for the Australian centre have been described elsewhere (Walters et al. 2000).

### Cleaning product exposure

Information on exposure to cleaning products at work and home was collected through the main questionnaire for ECRHS III. Participants recorded the frequency of use for ten common types of cleaning products at home. These were bleach; ammonia; stain removers or other solvents; acids (e.g. decalcifies, liquid scale removers, vinegar, hydrochloric acid); liquid or solid furniture polish or wax; furniture sprays; floor mopping sprays; glass cleaning sprays; and degreasing sprays such as oven cleaners. Additionally, three types of cleaning products were recorded for occupational exposure: alcohol, soaps or foams, and any other chemical product for disinfecting hands and other chemical disinfectants, e.g. glutaraldehyde, formaldehyde, chloramine-T, quaternary ammonium compounds. The full version of ECRHS III main questionnaires can be found online (European Community Respiratory Health Survey Group 2011).

All cleaning products were categorised into seven groups regardless of exposure location: bleach, all sprays, occupational chemicals, all polishes, ammonia, stain removers or solvents, and acids. Participants responding “yes” to any specific cleaning products were categorised as being exposed to that product type. In addition, frequent users of cleaning products were defined as participants who self-reported using any cleaning products on a weekly basis, either 1–3 days per week or 4–7 days per week. In contrast, infrequent users were defined as participants who self-reported using cleaning products less frequently, including never or less than 1 day per week.

### Asthma, COPD, and lung function

We defined current asthma by self-report of any episode of asthma or any asthma medication taken in the past 12 months. Lung function was measured with an EasyOne ultrasonic spirometer (NDD Medizintechnik AG, Zürich, Switzerland). Spirometry was repeated 15 min after 200 μg of salbutamol administered via a spacer. Forced expiratory volume (FEV_1_) and forced vital capacity (FVC) were recorded as the best of three manoeuvres, expressed as z-scores (Zock et al. 2007; Quanjer et al. 2012). COPD was defined as post-bronchodilator (BD) FEV_1_/FVC below the Lower Limit of Normal (LLN) (Global Initiative for Chronic Obstructive Lung Disease (Gold) 2022).

### Other variables

Occupation was recorded using a job questionnaire. Participants who reported being currently employed were coded according to the International Standard Classification of Occupations (ISCO-88) four-digit classification (International Labour Office 1990). Details on ISCO-88 coding are in the supplemental methods. A current smoker was defined as any smoking as of 1 month ago at the time of interview. The body mass index (BMI) was defined as the body weight (kg) divided by the square of the body height (m). Atopy was defined as an average Skin prick tests (SPTs) wheal diameter of 3 mm or greater for 1 or more of the allergens tested (Antunes et al. 2009).

### Statistical analysis

The LCA aimed to classify participants into mutually exclusive subgroups with similar patterns of cleaning product use (Rachid et al. 2024). Participants were assigned to the class for which they had the highest probability of membership. Details of LCA methods are given in the Supplementary material. The model fit characteristics for classes are in e-Table 1. To test whether the exposed profiles primarily reflected occupational exposure, we conducted a χ^2^ test comparing the proportions of participants engaged in high-exposure occupations across the four profiles.

The associations between LCA profiles and risk of asthma/impaired lung function were determined using multivariable regression. Potential confounders, chosen with reference to the literature and directed acyclic graphs (DAGs) (e-Fig. 1), were included in the final model (Greenland et al. 1999). For asthma, we adjusted for age, sex, occupation, BMI, and current smoking. For lung function, we adjusted for occupation and current smoking. For comparison, a standard multivariable regression was conducted for each cleaning product exposure and respiratory outcome. A sensitivity analysis was conducted by including only the randomly selected general adult population from ECRHS I (excluding the symptomatic sample). All analyses were performed using Stata for Windows (StataCorp, Stata Statistical Software: Release 14.2, College Station, TX, USA).

**Fig. 1:**
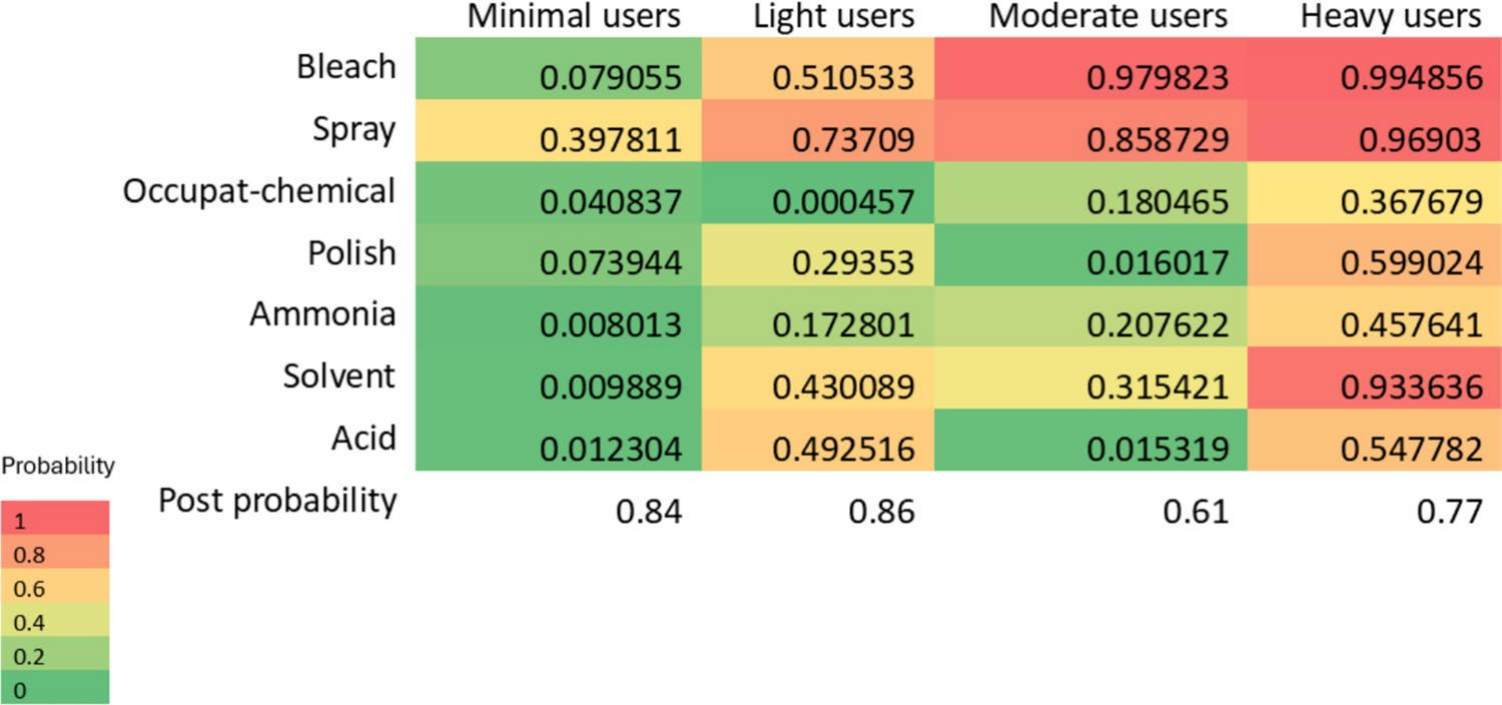
Heatmap of cleaning product classes determined by the latent class analysis (LCA)

## Results

Participant characteristics are presented in Table 1. Participants had a mean age of 55 years (standard deviation (SD) 6.2), and 28 (8.8%) reported current smoking. Current asthma and COPD were identified in 49 (15.6%) and 35 (12.9%) participants, respectively. Of the participants, 39 participants reported wheezing symptoms in the past 12 months without a previous diagnosis of asthma or COPD. The majority of participants (78.5%) were in mid to high-socioeconomic occupations.

**Table 1:** Characteristics of study sample (*N* = 318)

	Mean (SD) or *N* (%)
Age/years	55 (6.23)
Gender	
Male	147 (46.2)
Female	171 (53.8)
Current smoker	28 (8.8)
Former smoker	150 (47.2)
Atopy	
Yes	132 (52.6)
No	119 (47.4)
Occupations (ASCO skill level)	
Not currently employed	18 (6.0)
Elementary occupations	13 (4.3)
All workers/clerks/machine operators	98 (32.5)
Technicians	22 (7.3)
Professionals	117 (38.7)
Legislators/senior officials/managers	34 (11.0)
Current asthma	49 (15.6)
COPD	21 (7.6)

### Latent class exposure profiles

The four class LCA model provided the best fit based on the adjusted Bayesian information criterion and other fit parameters (e-Table 1). The four classes were labelled as “minimal users (reference)”, “light users (predominantly sprays)”, “moderate users”, and “heavy users” (Fig. 1). The prevalence and characteristics of cleaning product profiles are shown in Table 2. The most exposed profile, “heavy users” had a higher likelihood of using all cleaning products, whereas the least exposed profile represent participants who did not use cleaning products either at work or at home. There were no significant differences between the profiles for the proportions of participants engaged in high-exposure occupations (*P* = 0.95) (Table 2).

**Table 2:** Prevalence and characteristics of cleaning products identified by LCA

Profile name	Minimal users	Light users	Moderate users	Heavy users
Description	High probability that participants do not use cleaning products at work and at home	Higher probabilities of using bleach, sprays, solvents or acids. Occupational exposure was similar to reference group	Highest probability of using bleach and sprays, higher possibilities of using ammonia, solvents and occupational exposures	Most exposed group, highest probabilities of using bleach, sprays and solvents together, higher probabilities of using polish, ammonia, acids and occupational cleaning products
N (prevalence)	80 (25.4)	104 (33.0)	75 (23.8)	56 (17.8)
Male (%)	45 (56.3)	43 (41.4)	30 (40.0)	29 (51.8)
Current smoker (%)	6 (7.5)	8 (7.7)	9 (12.0)	5 (8.9)
Atopy (%)	40 (58.0)	39 (48.2)	33 (55.9)	19 (47.5)
Participants who engaged in high exposure occupation to cleaning products (%)	12 (15.0)	14 (13.5)	9 (12.0)	7 (12.5)

Although the frequency of product use could not be included in the LCA model, frequent use was more common in the “heavy users” profile compared with the “minimal users” (Table 3). Additionally, we conducted a sensitivity analysis including only the random participant sample (*n* = 239) (excluding the symptomatic sample). This did not alter the estimated associations between cleaning product profiles and respiratory outcomes (e-Table 3).

**Table 3:** The proportions of frequent users (at least once per week) across cleaning product classes

Types of cleaning products	Minimum users	Lighter users	Moderate users	Heavy users	*P* values
Bleach	0%	25.0%	42.7%	46.4%	< 0.001
Spray	5.0%	26.9%	29.3%	41.1%	< 0.001
Polish	1.3%	5.8%	0%	16.1%	< 0.001
Occupational chemicals	3.8%	41.4%	26.7%	53.6%	< 0.001
Ammonia	0%	3.9%	4.0%	12.5%	< 0.001
Solvent	0%	13.5%	12.0%	41.1%	< 0.001
Acids	0%	28.9%	0%	14.3%	< 0.001

### Cleaning product profiles and respiratory outcomes

**Fig. 2:**
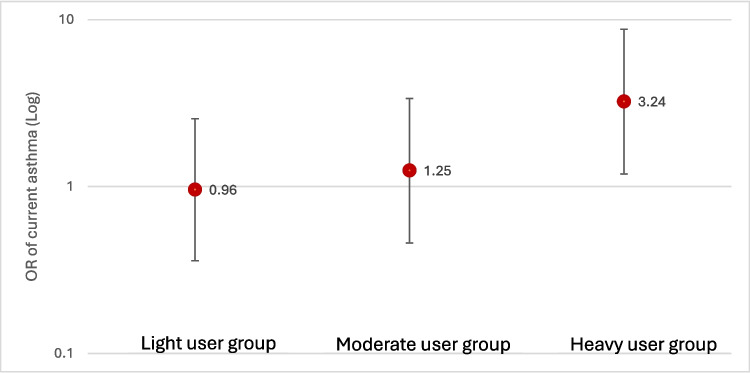
Adjusted association between cleaning product profiles and current asthma compared to the minimal user class

Belonging to the “heavy users” profile was associated with increased odds of current asthma compared with the “minimal users” (odds ratio (OR) 3.24, 95% confidence interval (CI) 1.19, 8.77) (Fig. 2). There were no strong associations for those belonging to the “moderate users” or “light users” profiles. We did not find any association between cleaning product profiles and COPD (Table 4).

**Table 4:** Adjusted ORs and coefficients* between cleaning products LCA and respiratory outcomes

Respiratory outcomes		Lighter users	Moderate users	Heavy users
Current asthma		0.96 (0.36, 2.55)	1.25 (0.46, 3.37)	**3.24 (1.19, 8.77)**
COPD		1.04 (0.30, 3.60)	0.90 (0.23, 3.61)	2.06 (0.55, 7.73)
Pre-bronchodilator (z-score)	FEV_1_	−0.14 (−0.45, 0.18)	−0.20 (−0.54, 0.14)	**−0.57 (−0.97, −0.17)**
	FVC	−0.17 (−0.46, 0.11)	−0.23 (−0.59, 0.02)	**−0.46 (−0.82, −0.11)**
	FEV_1_/FVC	0.06 (−0.21, 0.34)	0.08 (−0.22, 0.38)	−0.15 (−0.50, 0.19)
Post-bronchodilator (z-score)	FEV_1_	−0.01 (–0.32, 0.30)	−0.07 (−0.40, 0.27)	**−0.47 (−0.86, −0.07)**
	FVC	−0.15 (−0.45, 0.14)	−0.19 (−0.51, 0.12)	**−0.46 (−0.83, −0.08)**
	FEV_1_/FVC	0.24 (−0.05, 0.53)	0.19 (−0.13, 0.50)	0.04 (−0.33, 0.41)

^*^For current asthma outcome, association was adjusted for sex, age, occupations, BMI and current smoking; for lung function outcomes, associations were adjusted for occupation and current smoking

**Fig. 3:**
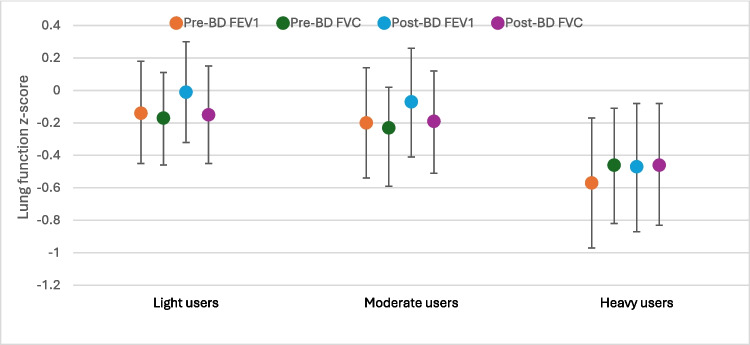
Adjusted association between cleaning product profiles and lung function as z-scores compared with the minimal user class

Belonging to the “heavy users” profile was strongly associated with reduced lung function parameters, including pre-bronchodilator FEV_1_ (β-coefficient for z-score: −0.57 [95% CI −0.97, −0.17]); FVC (−0.46 [−0.82, −0.11]); post-bronchodilator FEV_1_ (−0.47 [−0.86, −0.07]); and FVC (−0.46 [−0.83, −0.08]), compared with the “minimal users” profile (Fig. 3). Additionally, there were weak associations observed between the “moderate users” profile and pre- and post-BD FVC. No associations were observed for FEV_1_/FVC ratio.

### Individual cleaning product exposure and respiratory outcomes

Few associations were observed between individual cleaning products and respiratory outcomes (e-Table 2). Bleach was associated with increased odds of current asthma (OR 2.48 [95% CI 1.10, 5.63]), while solvents were associated with reduced pre-BD FEV_1_/FVC (β-coefficient for z-score: −0.27 [−0.53, −0.02]). Overall, no consistent trend emerged linking any specific cleaning product to respiratory outcomes.

## Discussion

Using comprehensive data from the Melbourne arm of ECRHS III, we found that exposure to cleaning products was associated with both current asthma and reduced lung function. There were particularly elevated risks observed among individuals who used multiple cleaning products on at least a weekly basis. To our knowledge, this is the first study to investigate respiratory health risks by integrating a comprehensive list of occupational and household cleaning products into innovative LCA-derived classes. These classes reflect real-world exposure patterns and provide meaningful implications for public health and workplace safety. Our findings support the development of detailed guidelines for using cleaning products, which may inform future asthma guidelines and risk reduction of accelerated lung function decline.

Risk reduction measures related to cleaning products for risk reduction and management of asthma are poorly identified in current guidelines (Global Initiative for Asthma 2024; National Asthma Council 2025). Although the Global Strategy for Asthma Management and Prevention 2024 recognised indoor air pollution as an avoidable risk factor for asthma management, it only focused on the toxic gases generated from cooking and heating (Global Initiative for Asthma 2024). Our results highlight the complex relationships between cleaning products and lung health, suggesting that the combined use of these products may have greater effects that were not captured when each class was assessed individually.

Growing evidence links cleaning products to increased asthma risk and lung function decline in adults. Analysis from the ECRHS and other cohorts has shown increased asthma risk and accelerated lung function decline associated with frequent household or occupational cleaning (Zock et al. 2007; Svanes et al. 2018; Da Silva et al. 2024). However, these studies generally investigated cleaning products individually, an approach that may have failed to capture combined effects. In contrast, our study revealed that participants in the most exposed profile (“heavy users”) had the highest respiratory health risks, exceeding the sum of individual effects derived from traditional regression analyses. Our novel methodological approach better captures the risks from real-life cleaning product use behaviour.

Exposure to cleaning products may cause airway irritation and chronic inflammation, which can subsequently lead to asthma symptoms and reduced lung function (Archangelidi et al. 2021; Dai et al. 2022). Understanding the ingredients in cleaning products is crucial for precisely identifying the mechanisms underlying these risks. Many commonly used cleaning products contain volatile organic compounds (VOCs) or other irritant chemicals, such as chlorine, ammonia, hypochlorite, hydrochloric acid and sodium hydroxide, and many have been linked to asthma, atopic dermatitis, allergies and reduced airway function (Steinemann et al. 2015; Clausen et al. 2020; Prasasti et al. 2021; Ha et al. 2022; Wang et al. 2023), particularly when inhaled as aerosols or sprays (Le Moual et al. 2014). Combined or repeated use of multiple products may further increase exposure to secondary pollutants formed through chemical reactions between the products in the home (Singer et al. 2006; Clausen et al. 2020). Unfortunately, the lack of detailed ingredient labelling and mandatory warnings on cleaning products limits consumer awareness of these potential risks (Welsh et al. 2000; Nicol et al. 2008; Environmental Defence 2017), especially when these products are combined, thereby potentially amplifying their harmful effects.

Our study did not detect a significant association between cleaning products and COPD. One possible explanation is that it is challenging to establish the long-term effects within a cross-sectional design, as COPD typically develops over many years. Several cohorts have reported links between long-term occupational cleaning exposure and COPD (Svanes et al. 2018; Dumas et al. 2019). Another possible reason is the small sample size, as only 21 COPD cases were detected. Inverse associations were observed for some individual products, possibly due to small sample sizes within each cleaning product exposure group. Reverse causation may also explain the results because people with asthma or COPD may avoid using these cleaning products.

Given increasing evidence of adverse impacts of cleaning products, consumers should carefully consider the necessity and frequency of use. Safer practices include using respiratory protection (e.g. face masks), ensuring effective ventilation during and after cleaning, avoiding using multiple products at the same time, and seeking out clearly labelled products (Dai et al. 2022; American Lung Association 2025). More needs to be known about individual vulnerabilities to respiratory harm e.g. infants and the elderly. We also recommend limiting the frequency of cleaning product use as much as possible. Evidence suggests that even increased use of “green” cleaning products is associated with a higher risk of uncontrolled asthma (Da Silva et al. 2024).

## Strengths and limitations

Using the ECRHS population-based and symptomatic samples with both surveys and spirometry, makes our results applicable to the general adult population. Our data enabled consideration of multiple cleaning products together and adjustment for potential confounders. We identified cleaning product profiles using LCA, which were able to model groups of products together. The combined use of these products may have additive or synergistic effects that were not captured when we assessed these exposures individually. The LCA approach captures real-life cleaning behaviour by modelling how cleaning products are typically used by individuals in the home and at work.

However, a major limitation of this study was its cross-sectional design, which raises the question of reverse causation. Individuals with pre-existing asthma could be more likely to use cleaning products frequently in an effort to reduce exposure to allergens or respiratory infections. Another weakness was the reliance on self-reported exposures to cleaning products, which may not accurately reflect actual exposure. This may have introduced misclassification resulting in bias towards the null. It is difficult and expensive to measure individual concentrations of cleaning products and directly capture differences in toxicity and concentrations of each chemical, so most observational studies rely on self-report. Another challenge is identifying potential secondary chemicals when cleaning products were mixed. The influence of secondary exposure is still not fully understood.

## Conclusions

Our study identified four cleaning product exposure classes based on LCA, which represented distinct exposure profiles, reflecting differences in types and frequency of use of cleaning products in real life. We found increased risk of asthma and lower lung function in adults belonging to the “heavy users” group who had the highest risks when compared to the “minimal users”. These findings highlight that combined and frequent use of multiple cleaning products may be associated with adverse respiratory outcomes in adults. Our findings support the need for public awareness of potential respiratory risks related to cleaning products and highlight the importance of considering safer and less harmful cleaning practices.

## Supplementary information

Below is the link to the electronic supplementary material.

ESM 1 (DOCX 210 KB)

## Data Availability

Due to the sensitive nature of the questions asked in this study, study respondents were assured raw data would remain confidential and would not be shared.

## Abbreviation

BD: Bronchodilator
BMI: Body mass index
CI: Confidence interval
COPD: Chronic obstructive pulmonary disease
COVID-19: Coronavirus disease 2019
DAG: Directed acyclic graph
ECRHS: European community respiratory health survey
FEV₁: Forced expiratory volume in 1 second
FVC: Forced vital capacity
GLI: Global lung function initiative
ISCO-88: International standard classification of occupations (1988)
LCA: Latent class analysis
LLN: Lower limit of normal
OR: Odds ratio
PM₂.₅: Particulate matter with aerodynamic diameter ≤ 2.5 µm
SD: Standard deviation
SPT: Skin prick test
VOC: Volatile organic compound

## Glossary

BD: Bronchodilator
CI: Confidence interval
COPD: Chronic obstructive pulmonary disease
ECRHS: European Community Respiratory Health Survey
FEV_1_: Forced expiratory volume in 1 s
FVC: Forced vital capacity
GLI: Global lung function initiative
LCA: Latent class analysis
LLN: Lower limit of normal
OR: Odds ratio
VOC: Volatile organic compounds
