# Supplementary material for: Cleaning products and classes associated with poor respiratory health
Source: Environ Sci Pollut Res Int. 2026 Mar 23;33(12):5485–93. doi: 10.1007/s11356-026-37616-z (PMC13091884; doi:10.1007/s11356-026-37616-z)

# Supplemental materials

## Supplemental Methods

**LCA methods:** The number of latent classes were examined sequentially starting at a 2-class model. The final number of latent classes was determined by model fit criteria (AIC, BIC and log-likelihood) by comparing models from 2 classes to 6 classes. This process identifies a best fit model which maximizes between-class and minimizes within-class variance, resulting in participants assigned to the class for which they had the highest probability of membership. Identified cleaning product features can then be related to the individual level behaviours of interest.

**ISCO-88:** Based on ISCO-88, five occupational skill levels were categorized as follows: “elementary occupations”, “all workers / clerks / machine operators,” “technicians,” “professionals,” and “legislators / senior officials and managers”. Participants who were not currently employed (including retired, unemployed, or homemakers) were classified into a separate “not currently employed” category.

Supplemental table 1 Model fit characteristics for 2-6 class models.

| No of class | Fit criteria | | | | | Class size | | | | | |
| --- | --- | --- | --- | --- | --- | --- | --- | --- | --- | --- | --- |
|  | Entropy | BIC | AIC | Adjusted BIC | Log likelihood | 1 | 2 | 3 | 4 | 5 | 6 |
| 2 | 0.60 | 210 | 154 | 162 | -1168 | 147 | 168 |  |  |  |  |
| 3 | 0.59 | 227 | 141 | 154 | -1154 | 95 | 157 | 63 |  |  |  |
| 4 | **0.63** | **251** | **135** | **153** | **-1143** | **80** | **104** | **75** | **56** |  |  |
| 5 | 0.63 | 290 | 144 | 167 | -1139 | 81 | 70 | 55 | 75 | 34 |  |
| 6 | 0.66 | 331 | 154 | 182 | -1137 | 81 | 78 | 40 | 68 | 34 | 14 |

Supplement table 2 Adjusted associations* for single cleaning product and respiratory outcomes (N=315)

| Cleaning products (N of exposed) | Current asthma | COPD | Pre-bronchodilator | | | Post bronchodilator | | |
| --- | --- | --- | --- | --- | --- | --- | --- | --- |
|  |  |  | FEV1 | FVC | FEV1/FVC | FEV1 | FVC | FEV1/FVC |
| Bleach (179) | **2.48 (1.10, 5.63)** | 0.80 (0.33, 1.91) | 0 (0.28, 0.27) | -0.06 (-0.31, 0.19) | 0.06 (-0.17, 0.30) | 0.06 (-0.21, 0.33) | 0 (-0.26, 0.25) | 0.08 (-0.17, 0.34) |
| Spray (225) | 0.53 (0.24, 1.17) | **0.34 (0.14, 0.81)** | 0.06 (-0.23, 0.35) | -0.07 (-0.33, 0.19) | 0.20 (-0.05, 0.45) | 0.03 (-0.25, 0.32) | -0.12 (-0.39, 0.14) | 0.24 (-0.03, 0.51) |
| Occupational chemicals (33) | 0.86 (0.29, 2.58) | 1.96 (0.52, 7.39) | 0.09 (-0.35, 0.54) | 0.18 (-0.22, 0.58) | -0.14 (-0.52, 0.24) | 0.06 (-0.38, 0.50) | 0.17 (-0.24, 0.59) | -0.15 (-0.57, 0.26) |
| Polish (77) | 1.61 (0.73, 3.56) | 1.61 (0.60, 4.33) | -0.19 (-0.51, 0.13) | -0.20 (-0.49, 0.09) | 0.05 (-0.22, 0.33) | -0.15 (-0.46, 0.16) | -0.14 (-0.43, 0.16) | 0.03 (-0.26, 0.33) |
| Ammonia (59) | 2.06 (0.92, 4.63) | 1.07 (0.37, 3.13) | -0.32(-0.67, 0.03) | -0.26 (-0.57, 0.06) | -0.11 (-0.42, 0.19) | -0.23 (-0.57, 0.12) | -0.19 (-0.51, 0.14) | -0.07 (-0.39, 0.26) |
| Solvent (124) | 1.40 (0.66, 2.99) | 1.78 (0.71, 4.48) | -0.23 (-0.52, 0.07) | -0.03 (-0.29, 0.24) | **-0.27 (-0.53, -0.02)** | -0.24 (-0.53, 0.05) | -0.10 (-0.38, 0.17) | -0.18 (-0.45, 0.09) |
| Acid (95) | **0.34 (0.14, 0.81)** | 0.55 (0.19, 1.60) | 0.03 (-0.27, 0.32) | 0.01 (-0.25, 0.28) | 0.01 (-0.25, 0.26) | 0.07 (-0.22, 0.36) | 0.01 (-0.26, 0.29) | 0.05 (-0.22, 0.32) |

*For asthma and COPD outcomes, all exposures were adjusted for age, sex, occupation, current smoker and other cleaning products use, for lung function outcomes, all exposures were adjusted for occupation, current smoker and other cleaning products use.

Supplement table 3 Adjusted associations* between cleaning products LCA and respiratory outcomes in the general population. (excluding the symptomatic sample)

| Respiratory outcomes | | Lighter users | Moderate users | Heavy users |
| --- | --- | --- | --- | --- |
| Current asthma | | 1.33 (0.22, 8.01) | **5.57 (1.03, 30.09)** | **5.21 (0.89, 30.57)** |
| COPD | | 1.41 (0.31, 6.33) | 1.15 (0.21, 6.20) | 1.37 (0.21, 9.03) |
| Pre-bronchodilator  (z-score) | FEV_1_ | -0.17 (-0.52, 0.18) | -0.24 (-0.61, 0.14) | **-0.41 (-0.86, 0.04)** |
|  | FVC | -0.17 (-0.49, 0.16) | -0.24 (-0.59, 0.11) | **-0.33 (-0.75, 0.09)** |
|  | FEV_1_/FVC | 0.01 (-0.29, 0.31) | -0.04 (-0.36, 0.28) | -0.11 (-0.49, 0.28) |
| Post-bronchodilator  (z-score) | FEV_1_ | -0.05 (-0.40, 0.30) | -0.08 (-0.45, 0.29) | **-0.36 (-0.82, 0.09)** |
|  | FVC | -0.16 (-0.49, 0.17) | -0.13 (-0.49, 0.22) | **-0.40 (-0.83, 0.04)** |
|  | FEV1/FVC | 0.24 (-0.05, 0.53) | 0.19 (-0.13, 0.50) | 0.04 (-0.33, 0.41) |

*For current asthma outcome, association was adjusted for sex, age, occupations, BMI and current smoker; for lung function outcomes, associations were adjusted for occupations and current smoker.

Supplement Figure 1 The proposed DAG with full adjustments.


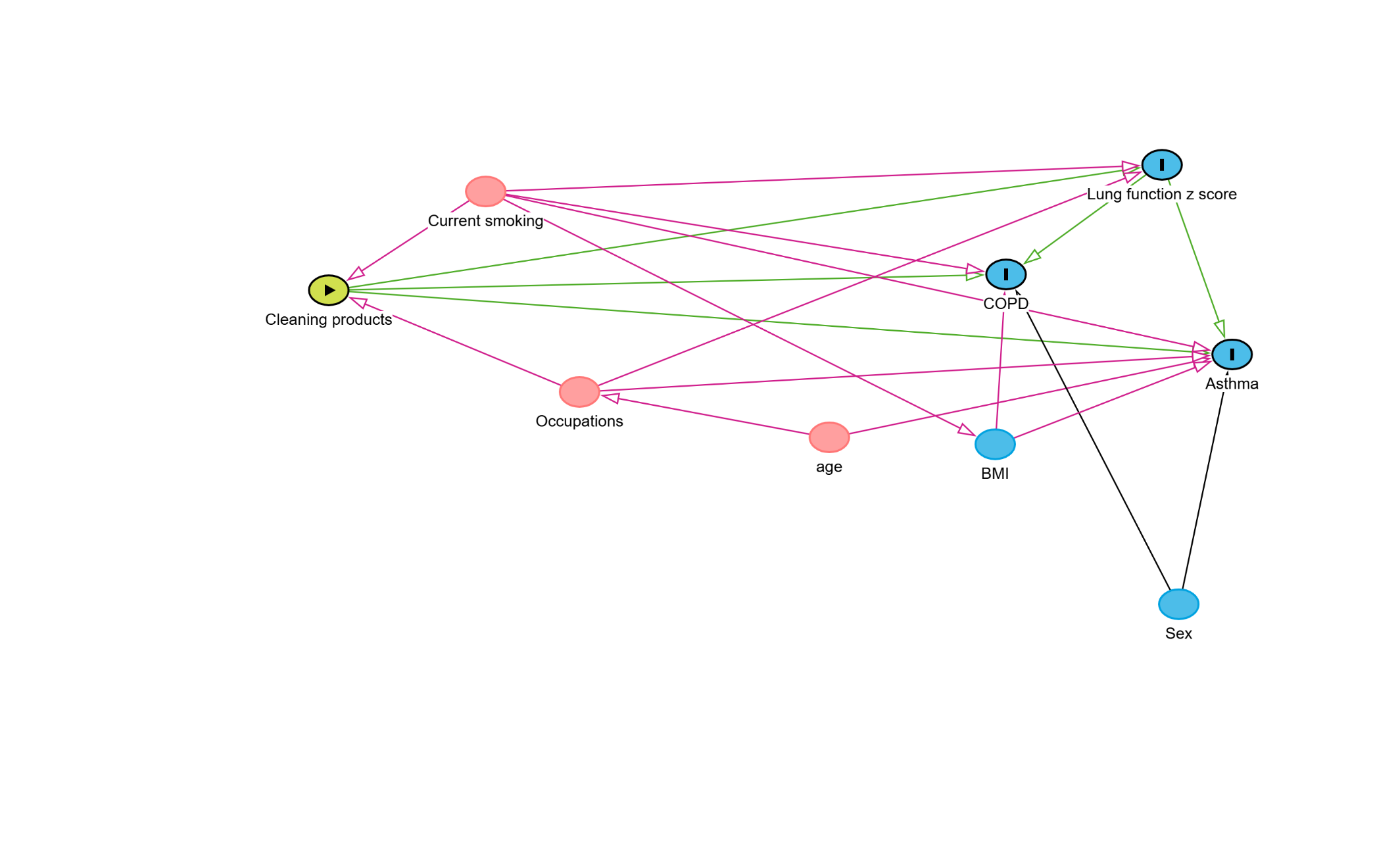

Supplement: Supplementary file 1 — (DOCX 210 KB) [file 11356_2026_37616_MOESM1_ESM.docx]
